# Clinical and Wear Analyses of 9 Large Metal-on-Metal Total Hip Prostheses

**DOI:** 10.1371/journal.pone.0163438

**Published:** 2016-10-06

**Authors:** M. C. Koper, N. M. C. Mathijssen, F. Witt, M. M. Morlock, S. B. W. Vehmeijer

**Affiliations:** 1 Department of Orthopaedics, Reinier de Graaf Hospital, Reinier de Graafweg 3, 2625 AD, Delft, the Netherlands; 2 Biomechanics Section, Hamburg University of Technology, Denickestraße 15, 21073, Hamburg, Germany; Harvard Medical School/BIDMC, UNITED STATES

## Abstract

**Background:**

Metal-on-Metal (MoM) total hip arthroplasties (THA) are associated with pseudotumor formation and high revision rates. This prospective study analysed the clinical and wear analyses of 9 large Metal-on-Metal (MoM) total hip arthroplasties (THA) to understand the underlying mechanisms of failure. The MoM bearings were revised for multiple reasons; the main reason was pseudotumor formation.

**Materials and Methods:**

From 2006 till 2010 the Reinier de Graaf Hospital implanted 160 large head M2a-Magnum™ (Biomet Inc. Warsaw, Indiana, USA) THAs in 150 patients. The first year, 9 bearings were revised and analysed at the Biomechanics Section, Hamburg University of Technology, Germany. We performed clinical (Harris Hip Score, radiographic analysis, blood cobalt and chromium) and wear analysis (implant, tissue and fluid) of the 9 bearings. Since this study did not fall under the scope of the Medical Research Involving Human Subjects Act in The Netherlands, no ethical approval was necessary. In this prospective study all patient details were anonymized by the corresponding author, all other authors were blinded during the research and wear analyses. Patients with bilateral MoM implants were excluded.

**Results:**

The 9 bearings had a median (IQR) survival of 41.0 (25) months in situ. From these bearings, three showed no noticeable wear. The median (IQR) head wear volume was 3.2 (3.6) mm^3^ and maximum wear depth 0.02 (0.02) mm. For the cup the median (IQR) wear volume was 0.23 (0.3) mm^3^ with a maximum wear depth of 0.03 (0.05) mm.

**Conclusion:**

An early identification of parameters related to failure of the MoM THA, such as pain, decreased range of motion, radiographic changes and high levels of blood cobalt and chromium is of great importance for patient’s quality of life. Especially now patients and surgeons face the long term effects of all these bearings still in situ. This study reports the clinical and wear analyses of 9 MoM THA. In the majority of this group the reason for revision was pseudotumor formation. Most bearings showed signs of wear, however with a great diversity in clinical analysis, in inclination angle, serum cobalt and chromium levels as well as wear analysis. For a better understanding of the underlying mechanisms related with failure, more wear analyses of revised MoM bearings are necessary as well as a frequent follow-up of the patients with a MoM bearing.

## Introduction

The Dutch Orthopaedic Association (Nederlandse Orthopedische Vereniging) decided in the beginning of 2011 to do a recall of all MoM articulations in The Netherlands. Their advice included active recall of all MoM hip implants as well as an active follow–up. This decision was made after multiple studies had shown high revision rates with the MoM THA by early failures and pseudotumor formation due to metal debris by high wear and edge loading [1–4]. In 2009 almost 35% of the 270.000 hip replacements in the USA were MoM bearings [1, 5]. In England, an estimated number of more than 60.000 patients have received a MoM THA since2003[6].

The metal debris can lead to elevated serum levels of cobalt and chromium and tissue reactions around the prosthesis, described as ‘Aseptic Lymphocyte dominated Vasculitis Associated Lesion’ (ALVAL), also known as pseudotumor [2]. The high wear and edge loading might be the result of suboptimal positioning or poor design of the components [7, 8]. Especially excessive inclination, with an inclination angle greater than 55 degrees as well as a small size of the cup, increases the edge loading. This edge loading might lead to high wear and local debris and is related to increased serum cobalt and chromium levels.[9, 10]. Furthermore, edge loading is proposed to have a relation to wear of modular taper interfaces [11].

The wear rates of the retrieved MoM bearings vary widely. First reports showed a low wear rate of 0.3mm^3^ per year [12, 13]. However, latest reports of MoM hip resurfacings and total hip prosthesis show high wear rates up to 6mm^3^ per year [14, 15].

This study describes the clinical and biological analysis of 9 patients with revised MoM THAs. We performed wear analysis of the 9 revised implants to relate the wear rate to our clinical and biological findings to help understanding the underlying mechanisms of failure.

## Materials and Methods

### Patient Demographics

In the Reinier de Graaf Hospital (Delft, The Netherlands) 160 primary large head MoM articulations were implanted in 150 patients between 2006 till 2010. Data and survival analysis of our cohort are written elsewhere [16]. From the 160 bearings placed in our facility, fifteen were revised of which two patients had bilateral MoM bearings. Thirteen of these implants were revised after the recall and 9 of these bearings were analyzed for this prospective study at the Biomechanics Section, Hamburg University of Technology, Germany. From these 9 bearings, seven were revised due to pseudotumor formation and in two cases progressive pain was the indication. Six of the patients were female and the mean age at primary surgery was 57 years (range 22–72 years). The median (IQR) time in situ was 41.0 (25) months. All components; cup, head and insert were revised, in one case the stem was also revised. The median (IQR) cup size was 52 (2) mm and head size 46 (2) mm. All patient demographic features are shown in Table 1. Patient no 9 had bilateral MoM THA, of which the right hip is revised.

**Table 1:** Patient demographics of the 9 revised bearings.

Ptn	Age	Gender	YoO	Indication	Headsize	Stem name	Pain	HHS	Diagnosis	Inclination angle(degrees)	Co levels(nmol/l)	Cr levels(nmol/l)	Histology
**1.**	57	F	2007	OA	46	Mallory	no	70>80	Tumor	47.69	22,9	18,4	Pseudotumor
**2.**	58	F	2007	OA	48	Taperloc	yes	60>70	Tumor/ Loosening	59.11	1431,2	510,9	Not performed
**3.**	60	F	2007	OA	46	Taperloc	yes	90>100	Tumor	59.44	80,9	67,4	Not performed
**4.**	65	F	2008	OA	46	Mallory	yes	60>70	Tumor	35.81	13,6	6,5	Not performed
**5.**	57	F	2009	OA	46	Taperloc	no	80>90	Tumor	52.07	80,8	100,1	Pseudotumor
6*	59	M	2009	AVN	56	Taperloc	yes	70>80	Loosening	32.49	22,1	35,9	Pseudotumor
7*	72	M	2009	OA	46	Taperloc	yes	<60	Pain	26.62	13,6	14	Pseudotumor
**8.**	22	M	2009	Pseudoarthrosis	46	Mallory	yes	70>80	Tumor	41.59	26,8	58,6	No clear diagnose
9**	67	F	2009	OA	48	Taperloc	no	90>100	Tumor	40.14	283,9	149,6	Pseudotumor

Patients

* marked had no pseudotumor.

** Bilateral MoM THA.

Abbreviations: Ptn = Patientnumber, YoO = Year of Operation, HHS = Harris Hip Score, Co = Cobalt, Cr = Chromium, OA = Osteoarthritis

Since this prospective study did not fall under the scope of the Medical Research Involving Human Subjects Act in The Netherlands, no ethical approval was necessary. In this study all patient details were anonymized by the corresponding author, all other authors were blinded during the research and wear analyses. Patients with bilateral MoM implants were excluded in measurements.

### Implants and operative technique

All patients received the Biomet Magnum (M2a-Magnum™) prostheses with Recap cup and Taperloc (Taperloc® Hip Stem) or Mallory stem (Mallory®) (Biomet inc. Warsaw Indiana, USA). The implants were implanted by two surgeons (one of the co-authors) and the selection of the type of implant was based on experience of the surgeon. Seven operations were performed through an anterior supine intermuscular approach and two (patient 1 and 8) through a straight lateral approach. During the first twenty four hours postoperatively antibiotic prophylaxis was given and patients received low-molecular-weight heparin for 6 weeks.

### Clinical analysis

All patients were examined clinically and asked if they experienced pain in the groin, suffer from deafness, dizziness, fear behavior/depression or experienced neurological problems after surgery. Additionally, the Harris Hip Score (HHS), a score to assess the results of hip replacement, and physical examination, all taken by one doctor, were used to evaluate all patients (S1 Fig). The HHS was divided into 5 categories (90 > 100 excellent, 80 > 90 good, 70 > 80 fair, 60 > 70 poor, <60 really poor) and used as one of our outcome measurements.

Anteroposterior pelvic and lateral hip radiographs were obtained and criticized by a specialized one radiologist. Radiographs were assessed for radiolucency, component migration, osteolysis and/or bone resorption. Lateral cup inclination was measured by two authors (MCK, NM) by using the transischial line and a second line drawn across the rims of the cup. Also, all patients had received an ultrasound exam of the hip by one specialized radiologist and additional MARS-MRI or CT-scan. Fluid components or mass on ultrasound or reactive masses on MARS-MRI /CT-scan were highly suspected for pseudotumor formation.

### Metal ion analysis

Blood was sampled from all patients in trace-element free tubes. Whole blood Cobalt (Co) and Chromium (Cr) levels (nmol/l) were measured by mass spectrometry (Atomaire Absorption Spectrometry, Thermo Elemental, Solaar M6, 2001, England). Advised by the Dutch Orthopeadic Association, the cobalt ranges were set as normal <40 nmol/l (< 2 mmg/L), normal high 40–85 nmol/l (2–5 mmg/L), high 85–170 nmol/l (5–10 mmg/L) and extreme high >170 nmol/l (> 10 mmg/L).

### Wear Measurement

**Fig 1:**
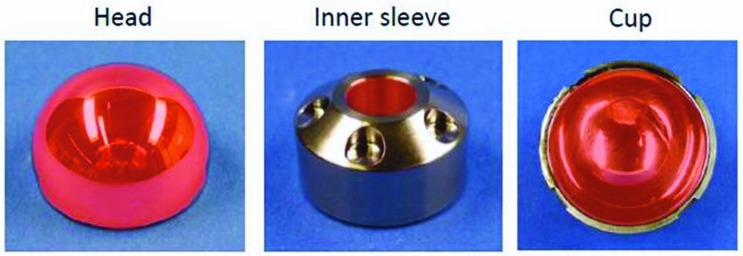
The red marked bearing and taper surfaces in the picture bellow were analysed.

Analysis of the bearings consisted of digital photographs and wear measurements. The surface geometry of each component was determined with the use of a coordinate measurement machine Mitutoyo BHN 305 (Mitutoyo Deutschland GmbH, Neuss, Germany). By using a 2 mm ruby tip all surfaces were scanned. The original surfaces were estimated by fitting unworn surfaces to regions of the measured surface which is unworn. For assessing bearing wear the geometrical from of a sphere was applied for fitting. Conical surface was calculated the same way, whereby the geometrical form of a cone was applied. Volumetric wear was quantified by comparison with an assumed initial geometry. Mathematical methods used are described in detail elsewhere [17, 18]. The red marked bearing and taper surfaces (Fig 1) were analysed.

### Tissue and Fluid analysis

During revision surgery, tissue and liquid samples around the joint of 6 patients were taken to determine cobalt, chromium and titanium concentrations. Samples were freeze-dried and crushed with a scalpel. 100 mg samples were digested by microwaves (ELAN DRC II and Optima7000DV ICP-OES, PerkinElmer, Inc. Waltham, MA, USA). Two samples were taken from the solution, separately analyzed and the results averaged.

## Results

### Clinical analysis

The mean Harris Hip Score was ‘fair’ (70 > 80). Patients revised because of pain scored a lower HHS (60>70) compared to the other revisions. Seven patients (77.8%) experienced pain in the groin, and 2 (22.2%) patients noted a swelling around the joint. None of the analysed patients showed neurological signs, signs of deafness or dizziness. Only one patient complained of fear during mobilisation which was related to the pain in the groin. Six patients complained of groin pain of which only four had pseudotumor formation. Three patients, all female, with pseudotumor tissue had no groin pain. This despite their serum increased ion level of cobalt and chromium and the wear in both cup and head.

### Radiographic analysis

The anteroposterior pelvic and lateral hip radiographs of patient 2 and 6 showed signs of loosening of the cup. All the other anteroposterior pelvic and lateral hip radiographs showed no signs of bone resorption, lysis or fractures. The lateral cup inclination had a median (IQR) angle of 41.2 (21.4) degrees. Patients diagnosed with pseudotumor formation scored a median (IQR) inclination angle of 47.7 (23.3) degrees. In our total cohort of 160 prostheses the mean (SD) inclination angle was 40.9 (7.3) degrees [16].

### Ultrasound analysis

Ultrasound was performed in 8 patients from our study group. Signs of pseudotumor were observed in 2 of these patients. Moreover, four patients showed liquid accumulation inside the joint or capsule. In one patient ultrasound was not performed, but CT and MRI was done immediately.

### Metal ion analysis

The median level of serum cobalt was 24.9 nmol/l with an interquartile range (IQR) of 65.15 nmol/l. For chromium a median of 47.3 nmol/l (IQR 76.8nmol/l) was found. Patients with a pseudotumor showed a median level of serum cobalt of 53.8 nmol/l (IQR 397.9nmol/l) and chromium of 63.0 nmol/l (IQR 187.4nmol/l).

### CT and MARS-MRI

A total of 6 MARS-MRI’s and 3 CT-scans were obtained of which 1 patient had both (patient no 9). In two cases CT-scan showed a possible pseudotumor. MARS-MRI showed in 5 cases a possible pseudotumor (see Table 1). CT-scan of patient number 7 showed no signs of pseudotumor. Patient no 6 had no CT or MRI investigation.

### Wear Analysis

**Fig 2:**
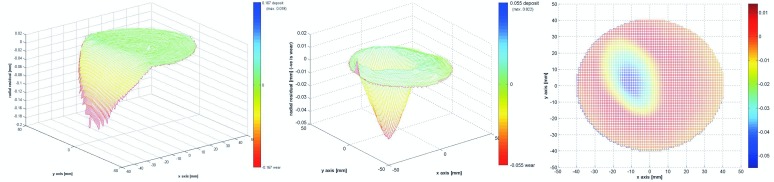
Wear plot graphs of the cup (most left) and head (middle and right) of patient no 2. 70% points used for estimation of wear. The green area represents the estimated original surface. The red area represents the wear and is defined as a negative deviation from the original surface.

Table 2 shows the wear analyses of all nine bearings. In three bearings no noticeable head wear was found. The median (IQR) head wear volume was 3.2 (3.6) mm^3^ and maximum wear depth 0.02 (0.02) mm. For the cup the median (IQR) wear volume was 0.23 (0.3) mm^3^ with a maximum wear depth of 0.03 (0.05) mm. Only one bearing, no 2, showed massive bearing wear. Notable cup wear of 28 mm^3^ and head wear 24.4mm^3^. Fig 2 shows the wear plots of this bearing. Clear inner sleeve taper wear was only seen in patient 9 (Table 2).

**Table 2:** Head, cup and taper wear in the revised bearings.

Ptn	Head wear			Cup wear			Inner Sleeve Taper wear		
	Wear Area Ratio [%]	Wear Volume [mm^3^]	Wear per Year [mm^3^]	Wear Area Ratio [%]	Wear Volume [mm^3^]	Wear per Year [mm^3^]	Deposits [mm^3^]	Wear Volume [mm^3^]	Wear per Year [mm^3^]
**1.**	6.382	1.209	0.2267	2.910	0.315	0.0591	0.078	0.000	0.000
**2.**	24.561	24.424	4.8047	20.575	27.998	5.5078	0.208	0.000	0.000
**3.**	13.042	4.486	0.9444	1.884	0.302	0.0636	0.002	0.000	0.000
**4.**	0.035	0.004	0.0009	0.000	0.000	0.000	0.041	0.000	0.000
**5.**	11.910	3.657	1.0703	0.000	0.000	0.000	0.118	0.000	0.000
6*	13.701	3.281	1.1931	0.000	0.000	0.000	0.266	0.041	0.0149
7*	0.000	0.000	0.000	1.825	0.178	0.0548	0.078	0.000	0.000
**8.**	6.484	0.930	0.310	2.302	0.260	0.0867	0.166	0.000	0.000
9**	9.210	3.204	1.1651	1.060	0.232	0.0844	3.074	0.172	0.0625

Patients

* marked had no pseudotumor.

** Bilateral MoM THA.

Abbreviation: Ptn = patientnumber

### Tissue and Fluid Analysis

The tissue and fluid analysis shows a great diversity between the bearings (Table 3). The largest difference is seen in the titanium tissue samples. The median (IQR) amount of titanium in the tissue is 168.5 (3327.2) mg/kg. Tissue analysis also showed a large amount of chromium with a great diversity between the samples. The median (IQR) of chromium was 6.9 (744.3)mg/kg. The other results are shown in Table 3.

**Table 3:** Tissue and fluid analyses of 6 revised bearings.

Ptn	Type	Co (mg/Kg)	Cr (mg/Kg)	Ti (mg/Kg)
**1.**	TissueFluid	44.3 1.11	29.8 0.77	1347<10
**2.**	Tissue Fluid	204 3.97	2945 26.37	9468<10
**3.**	Tissue Fluid	7.90 0.52	36.35 1.74	182 <10
**4.**	Tissue Fluid	5.34 <0.1	24.4 0.27	155 <10
**5.**	Tissue Fluid	9.6 91.56	48.7 4.24	<50 <10
7*	Tissue Fluid	15.6 0.13	37.5 0.27	<50 <10
Median (IQR)	Tissue Fluid	12.6 (77.0) 0.8 (2.0)	36.9 (744.3) 1.3 (9.5)	168.5 (3327.2) 10 (0)

Patient

* marked had no pseudotumor.

Abbreviations: Ptn = patientnumber, Co = Cobalt, Cr = Chromium, Ti = titanium

## Discussion

Survival of large MoM THAs has decreased by early failures and pseudotumor formation. Analysis of revised MoM THA shows a wide variation in wear rates and also pseudotumor formation in the absence of high wear [2, 4, 19]. A better understanding of failure, wear rate and clinical presentation is relevant to predict the outcome of MoM THA’s. We described the clinical and wear analysis of a small group of 9 MoM THA implanted in our clinic from 2007 till 2009. Seven prostheses were revised due to pseudotumor formation, two other prostheses because of pain and loosening.

Pseudotumors seem to be associated with high wear and metal hypersensitivity [20, 21], however a clear association has not been seen yet. According to Edward et al. the histopathological changes in the tissue cannot be explained by high wear alone [19]. There are also several reports of MoM THA failure and adverse local tissue reactions in patients with the absence of high wear [2, 4, 22]. In these patients a hypersensitivity reaction to the metal is more likely and results in aseptic lymphocytic vasculitis-associated lesions [23]. However, reducing the amount of wear might prevent this reaction and possibly reduce the formation of pseudotumors. This is also of importance for all other bearings. The most important predictor of wear rate is edge loading [24]. Edge loading is caused by high cup inclination, cup version, cup and head version, head-neck ratio, cup design and more variables. Some studies show excessive inclination, with an inclination angle greater than 55 degrees and a small size, increases the edge loading and might lead to high wear and local debris. An inclination of the acetabular component of more than 55 degrees is also related to increased serum cobalt and chromium levels [9, 10]. According to Hart et al, high cup inclination can even be a predictor of high wear rate [24]. The effects of metal wear particles and elevated serum metal have been documented but are still not understood [25]. High serum levels of cobalt and chromium were known and evolved during the running-in phase of the prostheses [26].

A clear correlation between the serum ion levels of cobalt and chromium and wear rate was not found in this study. This is in accordance with the findings of de Smet et al and Hart et al. [24, 27]. However, a trend in high inclination and increased metal ion levels could be observed (Table 1).

**Fig 3:**
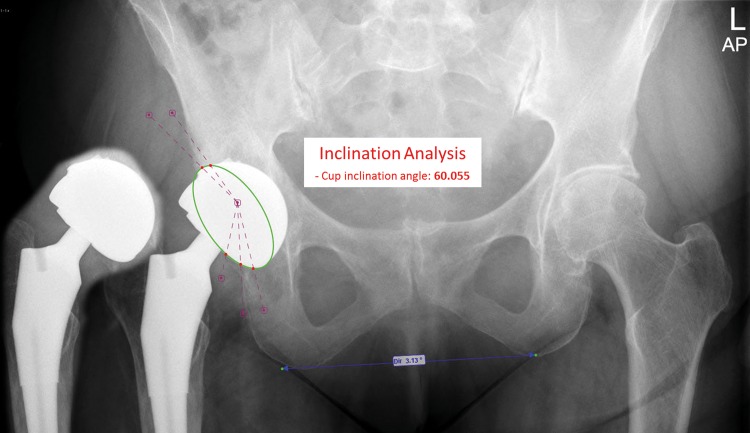
Anteroposterior radiograph of the pelvic from patient no 2 with the MoM total hip replacement on the right. The acetabular inclination angle was estimated 60 degrees and the anteversion angle 31 degrees.

In our small group, two patients (patient no 2 and 3) had a cup inclination angle over the 55 degrees. Both patients also show the highest cup wear area ratio (Table 2). However, high wear was also seen with an inclination angle of 47.7 and 32.5 degrees. The patients with high head wear ratio also showed an increased serum level of cobalt and chromium. Especially patient no 2 with an inclination angle of 60 degrees (Fig 3), a head wear ratio of 24.4 mm^3^ and a cup wear ratio of 28.0 mm^3^ showed highly increased cobalt and chromium levels (Table 1). These results show again the importance of a good positioned acetabular component especially to prevent the high edge loading and increase in serum and tissue ion levels as mentioned above.

The fluid and tissue analysis showed highly increased levels of cobalt and chromium in all patients. Thereby, even more striking is the high titanium level in the tissue of patient 1 and 2. These high levels of titanium indicate “trunniosis” in the taper-stem junction because only these two components consist of titanium alloys. Trunniosis, or cold-welding, is a phenomenon seen in the large head bearings, mostly above the 40mm [28–30]. More stress load on the modular interface at the larger head bearings implies more corrosion and debris, especially titanium. In our cases, no clear wear at the inner sleeve taper was found and stem wear analyses were not performed because the stem stayed in situ during the revision surgery. However, the large amount of titanium in fluid and tissue suggests wear at the taper-stem junction.

As shown above, the exact mechanisms for failure and pseudotumor formation are still not completely understood. Whether the failure is due to high cup inclination, edge loading, trunniosis, due to patient characteristics or a combination of all above, more wear analyses of revised bearings might help finding the answers. This study has several limitations. The clinical analyses as well as the wear analyses differs greatly. Furthermore, the gender ratio, age as well as the surgical technique varies within this small group. We also used two different stem types in our analysis. These sources may all bias the clinical and wear analysis and therefore limit the results of this study.

However, we can state all precautions should be taken for close monitoring and frequent control of MoM THA. Focussing on the clinical presentation can be misleading in decision making. Serum ion levels of cobalt, chromium (and titanium), radiographic control and MARS-MRI are all necessary for close monitoring. An example and flowchart for close monitoring and follow up is described earlier by our research group [16]. A better understanding of the process after implant placement in relation to clinical features, serum ion levels, pseudotumor formation and failure of the implant is necessary. Especially now we are facing the long term effects of the MoM THA’s still in situ.

## Supporting Information

S1 Fig Format of the Harris Hip Score. (PDF)
