# Supplementary material for: Clinical and Wear Analyses of 9 Large Metal-on-Metal Total Hip Prostheses
Source: PLoS One. 2016 Oct 6;11(10):e0163438. doi: 10.1371/journal.pone.0163438 (PMC5053776; doi:10.1371/journal.pone.0163438)
Supplement: S1 Fig — (PDF) [file pone.0163438.s001.pdf]

# Harris Hip Score

**I. Pain (44 points possible)**

| | |
|---|---|
| A. None | 44 |
| B. Slight, occasional, no compromise in activities | 40 |
| C. Mild pain, no effect on common activities, rarely moderate pain with unusual activity, may take simple pain medication | 30 |
| D. Moderate pain, tolerable, accepts limitations caused by pain. Some limitation of common activities or work. Occasionally takes pain medication stronger than aspirin | 20 |
| E. Pronounced, serious limitation of activities | 10 |
| F. Totally disabled, crippled, pain in bed, bedridden | 00 |

**II Function (47 points possible)**

A. Gait (33 possible)

| | | |
|---|---|---|
| 1. Limp | a. None | 11 |
| | b. Slight | 08 |
| | c. Moderate | 05 |
| | d. Severe | 00 |
| 2. Support | | |
| | a. None | 11 |
| | b. Cane for long walks | 07 |
| | c. Cane most of the time | 05 |
| | d. One crutch | 03 |
| | e. Two canes | 02 |
| | f. Two crutches | 00 |
| | g. Not able to walk | 00 |
| 3. Distance walked | | |
| | a. Unlimited | 11 |
| | b. 6 blocks | 08 |
| | c. 2-3 blocks | 05 |
| | d. Indoors only | 02 |
| | e. Bed and chair | 00 |

B. Activities (14 possible)

| | | |
|---|---|---|
| 1. Stairs | | |
| | a. Normally without using a railing | 04 |
| | b. Normally using a railing | 02 |
| | c. In any manner | 01 |
| | d. Unable to do stairs | 00 |
| 2. Shoes and Socks | | |
| | a. With ease | 04 |
| | b. With difficulty | 02 |
| | c. Unable | 00 |
| 3. Sitting | | |
| | a. Comfortably in chair for one hour | 05 |
| | b. On high chair for one half hour | 03 |
| | c. Unable to sit comfortably in a chair | 00 |
| 4. Enter public transportation | | 01 |

**III Absence of deformity point (4) are given if the patient demonstrated**

A. Less than 30° fixed flexion contracture
B. Less than 10° fixed adduction
C. Less than 10° fixed internal rotation in extension
D. Limb length discrepancy less than 3.2 centimetres

**IV Range of motion (index values are determined by multiplying the degrees of motion possible in each arc by the appropriate index**

| | | | |
|---|---|---|---|
| A. Flexion | | | |
| | a. | 0-45° | x 1.0 |
| | b. | 45-90° | x 0.6 |
| | c. | 90-110° | x 0.3 |
| B. Abduction | | | |
| | a. | 0-15° | x 0.8 |
| | b. | 15-20° | x 0.3 |
| | c. | Over 20 ° | x 0 |
| C. External rotation in extension | | | |
| | a. | 0-15° | x 0.4 |
| | b. | Over 15° | x 0 |
| D. Internal rotation in extension | | | |
| | a. | Any | x 0 |
| E. Adduction | | | |
| | a. | 0-15° | x 0.2 |

To determine the overall rating for range of motion, multiply the sum of the index values x 0.05. Record Trendelenburg test as positive, level or neutral.
